# Viability and Longevity of Human Miniaturized Living Myocardial Slices

**DOI:** 10.3390/jcdd12070269

**Published:** 2025-07-15

**Authors:** Ziyu Zhou, Yvar P. van Steenis, Surya Henry, Elisa C. H. van Doorn, Jorik H. Amesz, Pieter C. van de Woestijne, Natasja M. S. de Groot, Olivier C. Manintveld, Beatrijs Bartelds, Yannick J. H. J. Taverne

**Affiliations:** 1 Translational Cardiothoracic Surgery Research Lab, Department of Cardiothoracic Surgery, Erasmus Medical Center, 3015 GD Rotterdam, The Netherlands; z.zhou@erasmusmc.nl (Z.Z.); 499404ys@eur.nl (Y.P.v.S.); e.vandoorn@erasmusmc.nl (E.C.H.v.D.); j.h.amesz@erasmusmc.nl (J.H.A.); p.vandewoestijne@erasmusmc.nl (P.C.v.d.W.); 2 Unit Translational Electrophysiology, Department of Cardiology, Erasmus University Medical Center, 3015 GD Rotterdam, The Netherlands; n.m.s.degroot@erasmusmc.nl; 3 Department of Pediatrics, Division of Pediatric Cardiology, Erasmus Medical Center, Sophia Children’s Hospital, 3015 GD Rotterdam, The Netherlands; s.henry@erasmusmc.nl (S.H.); b.bartelds@erasmusmc.nl (B.B.); 4 Department of Cell Biology, Erasmus Medical Center, 3015 GD Rotterdam, The Netherlands; 5 Faculty of Electrical Engineering, Mathematics and Computer Sciences, Circuits and Systems, Department of Microelectronics, Delft University of Technology, 2628 CD Delft, The Netherlands; 6 Department of Cardiology, Erasmus Medical Center, 3015 GD Rotterdam, The Netherlands; o.manintveld@erasmusmc.nl

**Keywords:** living myocardial slices (LMSs), translational cardiology, experimental methods, in vitro models, 3D culture systems

## Abstract

Living myocardial slices (LMSs) have shown great promise in cardiac research, allowing multicellular and complex interplay analyses with disease and patient specificity, yet their wider clinical use is limited by the large tissue sizes usually required. We therefore produced mini-LMSs (<10 mm^2^) from routine human cardiac surgery specimens and compared them with medium (10–30 mm^2^) and large (>30 mm^2^) slices. Size effects on biomechanical properties were examined with mathematical modeling, and viability, contraction profiles, and histological integrity were followed for 14 days. In total, 34 mini-, 25 medium, and 30 large LMS were maintained viable, the smallest measuring only 2 mm^2^. Peak twitch force proved to be size-independent, whereas time-to-peak shortened as slice area decreased. Downsized LMSs displayed excellent contractile behavior for five to six days, after which a gradual functional decline and micro-architectural changes emerged. These findings confirm, for the first time, that mini-LMSs are feasible and viable, enabling short-term, patient-specific functional studies and pharmacological testing when tissue is scarce.

## 1. Introduction

Normal cardiac function relies on a complex interplay between numerous specialized cells within a tightly regulated electro- and biomechanical functional network. Such complexity is often simplified in various research models where oversimplified data hampers clinical translation [1]. Living myocardial slices (LMS) maintain a complex 3D architecture and overcome most of these limitations, bridging the gap between in vitro and in vivo research, potentially revolutionizing translational cardiac disease research [2,3,4,5]. LMSs are thin segments of living heart tissue, predominantly derived from surgical residual material or discarded transplantation hearts [1,3,6]. Given adequate electrical stimulation and defined preload conditions, these slices can exhibit sustained contractility lasting for up to four months allowing for patient- and disease-specific experimentation [5]. Importantly, LMSs retain a 3D multicellular structure with a close approximation of the in vivo cardiac environment, thereby acting as a valuable tool for experiments using patient-derived cardiac tissue [1,4].

Technology has evolved dramatically up to the point that patient-specific biomechanical analyses can be performed within hours after specimens are obtained from patients undergoing cardiac surgery. Several laboratories have previously shown that this biomimetic setup using LMSs for dedicated pharmacological testing is suitable and promising to accelerate pre-clinical testing [1,5,7,8].

However, tissue availability and the size of the available tissue form a major limitation for the widespread application of LMS. Traditionally, LMS protocols are confined to slices of around 8 × 8 mm, requiring initial tissue blocks of at least 1 cm^3^ [1,4,5,6,9]. Furthermore, using LMSs is technically challenging, and results depend very much on the skills of the operator [1,4,5,6,9]. Consequently, most current research on LMSs has been constricted to large specimens from explanted hearts and myectomy tissue, thus limiting the use of LMSs to adult cardiac pathology. The modification and adaptation of the current LMS setting towards the use of small specimens would greatly expand the field of in vitro biomechanical patient- and disease-specific testing and allow for a whole new research area in congenital cardiac disease and cardiac transplantation medicine. Up until now, it is unclear whether LMSs from very small specimens can even be cultured whilst remaining viable.

Compared to other 3D cardiac models such as organoids or engineered heart tissues, LMSs retain the native myocardial structure and function with minimal cell damage, offer high temporal and spatial resolution, and allow for the direct use of human biopsy tissue—suggesting their suitability for translational applications [10]. While the miniaturization of cardiac tissue platforms has been explored in other contexts, this study represents, to our knowledge, the first systematic attempt to apply LMS technology to very small specimens from patients.

As such, this study aims to (a) determine the feasibility of creating viable miniaturized LMSs, (b) examine the mathematical principles for down-scaling LMSs, (c) investigate biomechanical profiles across LMS sizes, and (d) assess the potential for the long-term culture of mini-LMSs.

## 2. Materials and Methods

### 2.1. Tissue Acquisition

Cardiac tissue was obtained from explanted hearts from patients undergoing cardiac transplantation, rejected hearts in lung transplantation, and myectomy tissue performed at the cardiothoracic department at Erasmus MC, Rotterdam, the Netherlands. In all cases, tissue was acquired from the left ventricle (LV). All patients consented to the use of surgical residual material for scientific research as approved by the medical ethics committee of the Erasmus MC, Rotterdam, the Netherlands (METC 2020-0988), and in accordance with local regulations and guidelines. Tissue specimens were immediately immersed in prechilled 4 °C Tyrode buffer (NaCl 136 mM, KCl 5.4 mM, MgCl_2_·6H_2_O 1 mM, NaH_2_PO_4_·H_2_O 0.33 mM, glucose 10 mM, CaCl_2_·2H_2_O 0.9 mM, 2,3-butanedione monoxime 30 mM, HEPES 5 mM, pH 7.4). The collected tissue was transported to our on-site laboratory where LMS preparation was performed within 24 h post excision.

### 2.2. LMS Preparation and Culture

**Figure 1:**
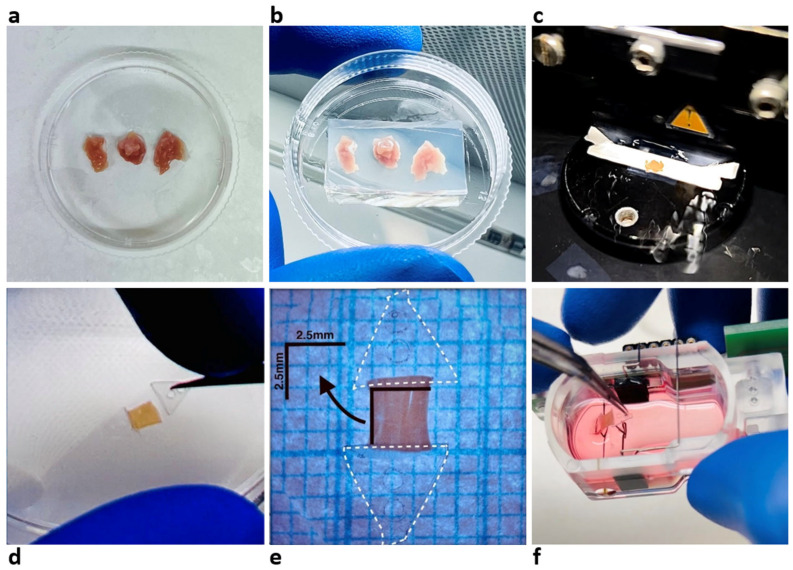
Mini-LMS preparation. (**a**) A representative sample of ventricular tissue obtained from a patient. (**b**) Ventricular tissue block embedded in agarose to ensure stabilization during slicing. (**c**) Tissue block mounted on a vibratome, ready for precise sectioning. (**d**) Triangular mounting devices (TMDs) used for holding and stabilizing the LMSs during cultivation. (**e**) Photographic record showcasing the orientation, size, and condition of the tissue slice post-sectioning. (**f**) A glimpse into the biomimetic cultivation chambers (BMCCs) housing the LMSs for their designated culture period.

Established protocols were adopted to produce LMSs [3,5,9,11]. In brief, the surgically obtained explanted tissue (Figure 1a) was trimmed into 1 × 1 × 1 cm tissue blocks and anchored with agarose to ensure stabilization on the vibratome stage (Figure 1b). The vibratome (VT1200 S, Leica BioSystems, Nussloch, Germany) was used to meticulously section the tissue to a thickness of 300 µm, using a speed of 0.07 mm/s and a vibration amplitude of 1.3 mm (Figure 1c). Once cut, slices remained in a cold-water bath at 4 °C until further preparation.

From the generated LMSs, mini-LMSs were produced by decreasing both the longitudinal and transverse dimensions. We defined three size categories: large (>30 mm^2^), medium (10–30 mm^2^), and mini (<10 mm^2^). LMSs under 1 mm^2^ were considered impractical given our current setup. After trimming the slices to a certain size, they were fixed to triangular mounting devices (TMDs) (Figure 1d). Next, the LMSs were submerged in 1.6 mL of 4 °C pre-cooled Tyrode buffer in a sealed Petri dish, measured using grid paper marked in 1 × 1 mm grids, and photographed using a 15× magnification digital camera linked to a microscope (Nikon D3100) (Figure 1e). Finally, the LMSs were cultured inside biomimetic cultivation chambers (BMCCs) (InVitroSys GmbH, Munich, Germany) (Figure 1f) filled with pre-warmed 2.4 mL medium (Medium 199 supplemented with Penicilline, ITS, and β-mercaptoethanol) and incubated at 37 °C with continuous recording of contractile force. All LMSs received electrical field stimulation at a current intensity of 50 mA, with a pulse duration of 3 ms and at a frequency of 0.5 Hz. All LMSs were mechanically stimulated under a diastolic preload of ca. 1 micro-Newton (mN), which is in-line with a near-physiological preload [5,6]. For prolonged culture, all LMSs received 1 µM isoprenaline, and stimulation frequency was set to 1 Hz to ensure prolonged contractility. The culture medium was renewed every other day with the addition of 1 µM isoprenaline.

### 2.3. Biomechanical Profile Measurement

**Figure 2:**
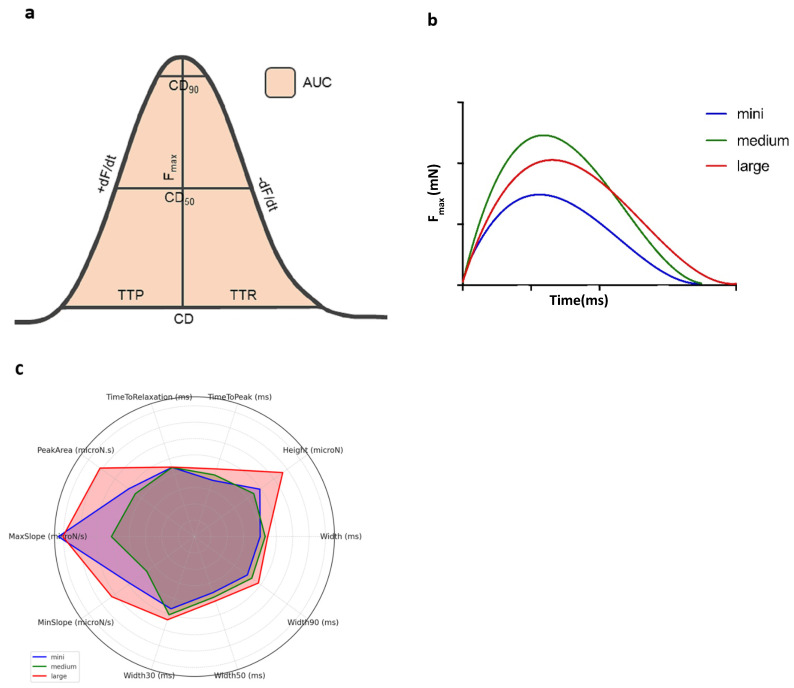
Baseline biomechanical profiles of mini-, medium and large LMSs. (**a**) Bell curve with the biomechanical characteristics of contractility: AUC = area under the curve. CD = total contraction duration. F_max_ = maximum contraction force. TTP = time-to-peak. TTR = time-to-relaxation. +dF/dt = steepest positive slope. −dF/dt = steepest negative slope. (**b**) Three contraction curves of nonlinear regression mean values for large, medium, and mini-LMSs (the colors represent large (red), medium (green), and mini-LMSs (blue)). (**c**) Radar plot of median values, specified by LMS size category (the colors represent the same categories as in (**b**)).

Approximately one hour after initiating the culture, 1.6 mL of medium was refreshed, and the preload was readjusted to ca. 1 mN. After another hour, and once contractile signals stabilized, the baseline biomechanical data of the LMS was gathered by averaging 30 s of contractions. Biomechanical parameters measured included maximum contraction force (*F_max_*, expressed in μN), contraction duration (CD, in ms), area under the contraction curve (AUC), and contraction up- and downslopes (dF/dt), including key timing metrics during contractions: time-to-peak (TTP) and time-to-relaxation (TTR), both in ms (Figure 2).

### 2.4. Mathematical Modeling and Statistics

To quantify the effect of miniaturizing *LMS size* on predicted *F_max_*, we constructed a linear regression model. The varying diseases underlying the *LMSs* were postulated to introduce variance, given the potential for confounding effects. Additionally, the experience of the researcher in producing *LMSs* was considered as another potential confounder due to the learning curve associated with *LMS* fabrication [5]. Both factors were included in the linear regression model to account for these potential confounders. The following is the basic model that we built:

$$F_{max}=\beta_{0}+\beta_{1}\times LMS\;size+\beta_{2}\times Disease+\beta_{3}\times Experience+\epsilon$$

To evaluate the model’s efficiency, we utilized various performance metrics, including a QQ-plot of standardized residuals and Cook’s distance. To analyze the contraction forces of mini-*LMSs*, we used a comprehensive statistical methodology with size normalization, including a log-normal distribution to correct for initial non-normality and a log transformation to approximate a normal distribution, enabling parametric testing. We selected our statistical model based on the Akaike Information Criterion (AIC), prioritizing both accuracy in our estimations and adherence to statistical parsimony. The initial model included the square root of *LMS size* (mm^2^), disease category (hypertrophic vs. non-hypertrophic), and operator experience as covariates. This results in the formula:

$$log(F_{max})=\beta_{0}+\beta_{1}\times \sqrt{LMS\;size}+\beta_{2}\times Disease+\beta_{3}\times Experience+\epsilon$$

Per size category (mini, medium, and large) and for each biomechanical parameter, the median and interquartile range (IQR) were calculated. For each parameter, a Kruskal–Wallis H test was used to determine the statistical significance between mini-, medium, and large *LMSs*. Subsequently, we performed Dunn’s Test for post hoc analysis and applied Bonferroni correction to control for multiple comparisons. Furthermore, for the parameter *F_max_*, we tested a continuous correlation with *LMS size* by using Spearman’s Correlation test. Given that neither the *F_max_* output nor the *LMS size* can be negative, we hypothesized that the *F_max_* produced corrected by LMS size (*F_max_*/mm^2^) followed a log-normal distribution. Thus, we tested the natural logarithm-transformed, size-corrected maximum force (ln(*F_max_*/mm^2^) for normality through both visual data assessments (histograms and QQ-plots) and statistical testing (Shapiro–Wilk normality test).

All stored data was extracted into LabChart 8 Pro software (Ad InstrumentS, Dunedin, New Zealand) for biomechanical analyses. For statistical analyses, we used R Statistical Software (v4.1.1; R Core Team 2021). IQRs were calculated for all biomechanical parameters. A *p*-value below 0.05 was deemed statistically significant.

### 2.5. Histology of Prolonged Culture

LMSs (*n*  =  16) from two patients were used for prolonged culture with histological analyses at various stages of cultivation (days 0, 5, 10, and 14). The LMSs were rinsed in phosphate-buffered saline (PBS) before being fixed in 4% paraformaldehyde (PFA) solution overnight, followed by three extensive PBS washes to remove any residual fixative. The fixed slices were stored at 4 °C until further use.

### 2.6. Paraffin Embedding

After fixation, LMS were dehydrated through a graded ethanol series (70%, 80%, 90%, 96%, and 100%), cleared in xylene, and embedded in paraffin using standard procedures. Paraffin-embedded samples were sectioned at 5 μm thickness using a microtome and mounted on glass slides for subsequent staining.

### 2.7. Immunohistochemical Staining

Hematoxylin and eosin (HE) staining was performed on the HE-600 (Ventana Medical Systems Inc., Oro Valley, AZ, USA). Tissue sections were deparaffinized using xylene, rehydrated through 100% ethanol, and then immersed in water. For nucleus staining, the tissue was treated with hematoxylin (Roche, Basel, Switzerland), followed by 25% ammonia water. For the background, tissues were incubated with eosin (Roche), subsequently dehydrated through increasing ethanol grades (96%, 100%), and cleared using xylene. The prepared slides were then covered with coverslip Activator (Roche).

Sirius Red Staining. Tissue sections were deparaffinized using xylene, rehydrated through decreasing grades of ethanol, and then brought to water. The slides were immersed in a Sirius Red solution for 1 h, washed with 0.5% acetic acid, dehydrated through increasing ethanol grades, and cleared with xylene. The treated slides were then covered with an anti-fading medium.

The immunostaining of the LMSs was performed on 5 µm thick whole-slide sections from formalin-fixed, paraffin-embedded (FFPE) tissue blocks, using the Ventana Benchmark Discovery (Ventana Medical Systems Inc., Tucson, AZ, USA). The tissue sections were deparaffinized in xylene (Sigma, Cat no. A5597-1GAL, Livonia, MI, USA) and dehydrated with reducing % of EtOH (VWR, Cat no. 83813.360, Radnor, PA, USA). The sections were then permeabilized in pre-boiled TE buffer pH 9.0 for 5 min and then cooled in ice water for 30 min before blocking the sections in 3% BSA (VWR, Cat. no. 422351S). The sections were then incubated with primary antibodies, namely, anti-sarcomeric alpha Actin antibody (Abcam, Cat no. ab137346 & ab9465, Cambridge, UK), CX43 (Abcam, Cat no. ab11370), and anti-Actin (#760-2601, Cell Marque, Rocklin, CA, USA), overnight at 4 °C. The slides were then incubated in relevant secondary antibodies (1:1000 dilution), namely, anti-mouse and anti-rabbit conjugated with either 488 or 647 Alexa Fluor® dyes (Molecular Probes) together with Hoechst 33258 (1:2500 dilution, Invitrogen, Cat no. H3569, Carlsbad, CA, USA), for 1 h in dark conditions. Finally, the slides were covered with an anti-fading Prolong Diamond antifade mountant (Invitrogen, P36961).

Fluorescence signal intensities were quantified using ImageJ (version 1.53t, National Institutes of Health, Bethesda, MD, USA) software by selecting the appropriate color channels for each marker, such as the red channel for Sirius Red staining and the green channel for α-SMA immunostaining. For each timepoint (day 0, 5, 10, and 14), regions of interest were defined based on signal localization or predefined areas. The extracted intensity values were exported to GraphPad Prism 8 for statistical analysis. Two-tailed unpaired t-tests were used to compare each timepoint to baseline (day 0). A *p*-value less than 0.05 was considered statistically significant.

## 3. Results

A total of 89 LMSs were generated from 13 patients, resulting in 34 mini-LMSs, 25 medium LMSs, and 30 large LMSs. Underlying disease included two patients with hypertrophic cardiomyopathy who underwent surgical myectomy, eight adult patients with end-stage dilated cardiomyopathy who underwent heart transplantation, and one pediatric patient with dilated cardiomyopathy who underwent heart transplantation. Two additional “healthy” hearts were used from lung donor patients that were not allocated to cardiac transplant. The sizes of the LMSs ranged from 2 mm^2^ to 56 mm^2^, with a median size of 15.0 mm^2^ (IQR: 7.47–42 mm^2^).

### 3.1. Statistical Model and Biomechanical Profiles Across Groups

The final regression model predicting log-transformed *F_max_* was:

$$\log\left(F_{\max}\right)=6.87+0.14\;\times \;\sqrt{\mathrm{LMS}\;\mathrm{size}}-0.29\;\times \;\mathrm{Disease}$$

In our model, the estimated coefficient for the covariate of *LMS size* was 0.015 ± 0.006, which was statistically significant (*p* = 0.02). The back-transformation of the coefficient resulted in a coefficient of 1.015 (95% CI [1.003, 1.027]), indicating a one-unit change of *LMS size* (i.e., a 1 mm^2^ increase in size), which increased the expected contraction force by approximately 1.5%. Neither the coefficient for disease type nor that for expertise effect significantly impacted the estimated contraction force (*p*-values of 0.352 and 0.271, respectively).

Table 1 summarizes the baseline biomechanical profile parameters by size category (mini, medium, and large). Among all parameters evaluated to characterize contractile properties, only TTP was significantly shorter (*p* = 0.003) in mini-LMSs, with a 9.7% increase in medium slices and up to 20.4% increased TTP in large LMSs. All other parameters showed no significant differences between the three groups with consistent F_max_, where contractile profiles between mini and medium slices were the same. Given the generalizable biomechanical profiles between those two groups, all further analyses will incorporate mini- and medium LMSs as one group. Figure 2 provides a visual summary of the baseline biomechanical profile across the groups.

### 3.2. Biomimetic Culture and Viability of Mini-LMS

**Figure 3:**
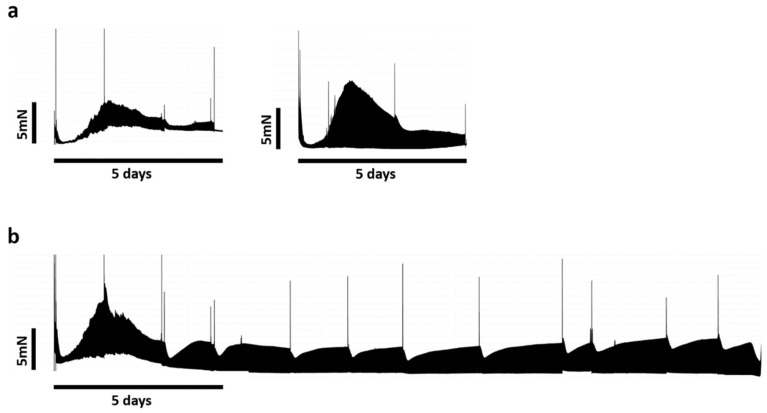
Continuous contractility recording. Positive spikes of contractility were produced by isoprenaline administration and corresponded to medium exchange intervals (36–48 h). (**a**) Contractility from mini-LMSs from two different patients. (**b**) Contractility from large-LMSs from first patient.

The contractile force pattern from two different patients over time is depicted in Figure 3, showing a comparison between the semi-acute mini-LMS profile of five days versus a prolonged large LMS culture. An initial decline in contractility was observed between 6 and 24 h post-incubation, followed by a recovery to peak levels within the subsequent 24–36 h. Beginning at 48 h, a gradual decrease in contractile force was noted, which stabilized by day 3. Specifically, for mini-LMSs, the contractile force remained stable for four–five days; thereafter, there was a baseline increase, and smaller contractions were seen by day 5–6. For the ensuing week, mini-LMSs could be stimulated; however, contractile profiles were dampened, only allowing for short contractile peaks upon pharmacological stimulation. This effect was short-lived but was present until day 14.

### 3.3. Micro-Architectural Organization and Cellular Morphology

**Figure 4:**
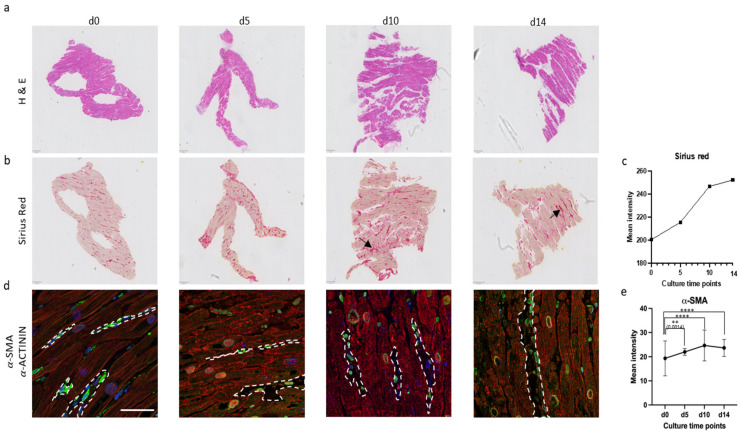
Histology analysis of cultured slices. (**a**) Myocardial tissue architecture after long-term culture (2 weeks) was monitored by hematoxylin and eosin (H & E) staining. (**b**) Sirius Red staining was performed for tissue fibrosis (indicated in black arrow). (**c**) Quantification of Sirius Red intensity showed increased fibrosis at days 5, 10, and 14 compared to baseline (d0). Representative image shown (*n* = 1). (**d**) Immunostaining analysis of *α*-SMA (green) and *α*-ACTININ (red) revealed increasing myocardial fibrosis in cultured slices (highlighted in white). (**e**) Quantification of mean *α*-SMA intensities in d0, d5, d10, and d14 showed elevated levels of *α*-SMA in d10 and d14 slices compared to baseline (d0). Data represent mean ± SD from five slices per timepoint (*n* = 5). Abbreviations: H & E—hematoxylin and eosin; *α*-SMA—alpha-smooth muscle actin; *α*-ACTININ—sarcomeric alpha-Actinin. Statistics: Data in (**c**,**e**) were analyzed using two-tailed unpaired *t*-tests comparing each timepoint to d0. Asterisks indicate significance: *p* < 0.01 (**); *p* < 0.0001 (****).

Baseline control (d0) showed well-organized cardiac muscle bundles with minimal signs of fibrosis. After five days, mini-LMSs displayed a reduced presence of eosinophilic staining, indicating varying amounts of collagen deposition, which is a sign of elevated fibrotic activity (Figure 4). To further quantify collagen levels, Sirius Red staining was performed. The baseline exhibited a limited deposition of collagen, represented by pale red staining. The d5, d10, and d14 slices demonstrated an increase in Sirius Red-positive areas compared to the baseline, confirming elevated collagen levels and enhanced fibrotic activity (Figure 4b,c; black arrow).

Immunohistochemical analysis targeting α-SMA and α-ACTININ was performed to further assess myofibroblast activation as a key indicator of fibrosis and sarcomere organization, respectively. The baseline slice exhibited a minimal number of α-SMA-positive cells and striated organization of α-ACTININ compared to d5, d10, and d14, indicating normal levels of myofibroblasts amidst cardiac cells containing a proper alignment of sarcomeric units in d0 samples. Conversely, slices cultured up to d5, d10, and d14 displayed a significant upregulation of α-SMA and disorganized α-ACTININ signals, suggesting disrupted sarcomeric architecture and an increased presence of myofibroblasts (Figure 4d,e; highlighted in white).

**Figure 5:**
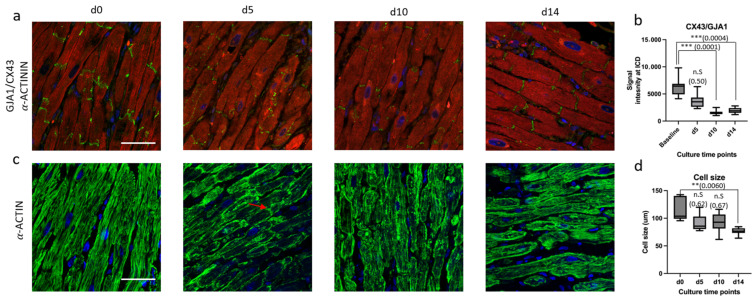
Immunostaining analysis of cultured slices (**a**) CX43/GJA1 (green), *α*-ACTININ (red), and Hoechst 33342 (blue) staining was performed to visualize proteins at intercalated discs (ICDs), striated sarcomere, and nuclei of myocardial cells. (**b**) Quantification of signal intensity measurements of CX43/GJA1 at ICDs showing decreasing intensities in slices cultured longer, i.e., d5, d10, and d14, when compared to baseline (d0). Data represent mean ± SD from five slices per timepoint (*n* = 5). (**c**) Immunostaining analysis of *α*-Actin (green), showing irregular *α*-Actin stainings in d5, d10, and d14 when compared to baseline (d0). *α*-Actin was also seen at ICD in d5 slice (indicated in red arrow). (**d**) Quantification of cell length in d0, d5, d10, and d14 using CX43 staining (green) in A as cell boundaries revealed decreasing cell length in d5, d10, and d14 slices compared to baseline (d0). Data represent mean ± SD from five slices per timepoint (*n* = 5). Abbreviations: CX43—Connexin 43; *α*-ACTININ—sarcomeric alpha-Actinin; ICDs—intercalated discs; *α*-ACTIN—alpha Actin. Statistics: Data in (**b**,**d**) were analyzed using two-tailed unpaired t-tests comparing each timepoint to d0. Asterisks indicate significance: *p* < 0.01 (**); *p* < 0.001 (***).

To further asses the integrity of intercalated discs and the structural organization of Z-discs, immunofluorescent staining for CX43 and α-ACTIN was performed, respectively. There was a significant reduction in intercalated disc signal intensity after day 10 (Figure 5a,b). At baseline, a regular and striated pattern of α-ACTIN was observed, reflecting the proper alignment of sarcomeric units. After day 5, α-ACTIN stainings exhibited a disorganized and irregular pattern, indicating structural abnormalities in the sarcomeric organization with consequential significant reduction in cell size as compared between baseline and day 14 (Figure 5c,d).

## 4. Discussion

Here, we confirm the feasibility of producing viable mini-/medium-sized LMSs from smaller tissue specimens, showing excellent contractile profiles for the first five to six days. Force generation, as assessed by overall force dynamics and contractile profiles, was comparable across groups. While most parameters showed no significant differences, we observed a minor variation in one time-related parameter, time-to-peak (TTP), which nonetheless remained within a narrow and physiologically consistent range across all groups. However, the ensuing culture showed a rapid decline in biomechanics, with consequential micro-architectural changes for up to 14 days, which are most likely attributable to the vulnerability or altered force distribution of mini-LMSs. Nevertheless, our data shows that the semi-acute experimentation of small-sized specimens is indeed feasible and the interpretation of contractile data is generalizable between groups, providing a valuable novel in vitro testing platform within the field of biomimetic research for congenital cardiac disease and transplantation medicine.

### 4.1. Statistical Modeling and Biomechanical Profile Across Groups

Given the inherent biological variation in the biomimetic culturing of LMSs, we first wanted to analyze whether the culture and loading conditions of large LMSs could be extrapolated to the mini-LMS setting. This method ensured a solid foundation for our analysis of biomechanical profiles of mini-LMSs, revealing no significant differences between mini and medium-sized groups across all parameters upon downsizing LMSs from medium to mini. This reflects a standardized contractile profile for LMSs between 10 and 30 mm^2^ with a maintained force generation, which was rather unexpected. One would imagine that the amount of force produced is dependent on size, which was not the case. However, during downsizing, the time needed to create this same amount of force reduced, as shown by a decrease in TTP as the LMSs got smaller. Contrarily, its counterpart TTR remained similar in the setting of a non-significant change in contractile force. There is around a 10% decrease in TTP to achieve about the same contractile force in the mini- and medium-sized LMSs, resulting in a consequential decrease in width of the contractile curve. Within the current setup and given the same force generation with a constant preload of ~1 mN, it is uncertain whether this sole variable of time-to-peak (and its derivative width) is an effect of downsizing rather than muscle-to-muscle variation. Indeed, Janssen’s group previously analyzed mouse trabeculae in a different setup and found that the speed and timing of contraction in the isolated myocardium is directly correlated to the speed and timing of relaxation, with a deviation of about 10% [12]. Previous data already suggested that sarcomeric length, stimulation frequency, and β-adrenergic stimulation are the main regulatory systems to change contractile force, dependent on Ca^2+^ sensitivity [13,14], placing the governing mechanics of contraction and relaxation within the sarcomeric matrix [12]. Nevertheless, they showed that, in different subsets of murine strains, there is no difference in the first derivative of force development over time under various conditions of frequencies and preloads [12]. Despite a constant LMS size in that study, our experiments show that using a normalized preload of 1 mN has a similar effect on force despite the differences in size. In terms of adjusting the preload in smaller LMSs, a recent study using whole-heart mouse LMSs showed no differences in force generation between 1.2 mN compared to 0.3 mN, corroborating the standardized use of 1 mN in our study [15]. Given that the determination of the optimal preload conditions was not within the scope of this study, the higher preload conditions could have been responsible for relatively fast histological changes after one week. Nevertheless, as we chose not to add an extra variable (the adjusted preload), some inconsistencies still exist on the optimal loading conditions [6,16,17]. As shown in the original viability study by Dendorfer, there is a linear relationship between shortening and contraction force, and given the fact that we show no biomimetic differences between mini- and medium LMSs [6], it is feasible to say that the biomechanical profile interpretations of standardized LMSs can be extrapolated to the miniaturized setting.

### 4.2. Biomimetic Culture of Mini-LMSs and Structural Changes

All mini-LMSs showed good contractile profiles for up to five to six days. Afterwards, an upwards baseline drift occurred with significantly decreased contractions for the ensuing period. Although contractile force resurged after isoprenaline administration for up to two weeks, this effect was diminished and short-lived for the prolonged culture, suggesting that mini-LMS experiments are best performed within that first week of culture. Histological examination results possibly substantiate these biomechanical alterations over time, where a significant increase in fibrotic tissue is seen after around five to six days in culture. On day 14, a 1.5-fold increase in fibrosis was noted as compared to the baseline from d0. Previous research showed that constant stretch should suppress fibroblast proliferation in LMSs [6,18] and that despite the expression of several growth factors and matrix proteins, no overt fibrosis or myocyte remodeling was seen during weeks of culture [6]. Contrary to those initial results, our study does show that mini-LMSs exert fibrotic changes after approximately six days, with concomitant changes in biomechanical profile, aligning with previous results using rabbit LMSs or stem cells in culture [16]. It is well established that sarcomere lengthening is related to contraction force [16], whereby lengthening up to a certain point results in an increased contractile capacity, after which it decreases again. Contrary to our initial expectations that mini-LMSs would generate a lower force, our study shows that the ability to generate the same amount of force is possible independent of LMS size, conditional on the same amount of preload. In order to further address micro-architectural changes, additional histology was performed.

Alpha-Actinin is an essential cytoskeletal protein that plays a vital role in cross-linking actin filaments and maintaining micro-architecture and the stability of the cytoskeleton. Compromises in their structure can lead to impaired cell function and altered biomechanics. The staining results of alpha-Actinin showed a gradual shortening of myocardial sarcomere length over time, which also explains why the baseline is increasing, as previously shown [19]. Nevertheless, Chabanovska’s group [19] already showed a significant reduction in alpha-Actinin by day 5, as opposed to our findings, where the gradual decline becomes significant by day 16. In addition to that, one of the critical mechanisms of cardiac remodeling is the change in intracellular electrical coupling caused by the aberrant expression of Cx43 [20,21], where Cx43 is considered to be a target of Wnt/beta-catenin transcription which interacts with β-catenin [20,22]. Despite continuous stimulation, a significant decrease in the expression of GJA1/Cx43 was observed from day 10, representing cardiac remodeling. Contrary to our results, the study of Watson previously showed a maintained Cx43 expression when normal LMSs were cultured with electrical stimulation [16] and a similar reduced expression in unloaded LMSs. They also reported no reduction in GJA1 expression in both loaded and unloaded conditions. However, similarly to our results, a recent study, using an air–liquid interface, which was readily used before the emergence of the electromechanical stimulation of slices, also showed that electrical impulse propagation was maintained for the first week and began to decrease by day 10 in culture [17].

### 4.3. Future Perspectives

In contrast to smaller cardiac slices from other labs derived from non-human mammalian hearts through a whole-heart slicing approach, our mini-LMSs are uniquely developed from tiny human tissue samples. Producing human mini-LMSs in an isotonic biomimetic system, to our knowledge, has not been performed elsewhere. Although murine heart slices are typically small, at about ±10 mm^2^, these slices are cross-sectional cuts of the heart, thus representing different contractility compared to these human LMSs, which are made from uniformly arranged fibers. The use of mini-LMSs presents a unique opportunity as they have preserved multicellularity and extracellular matrix, allowing for patient-specific translational studies. Better yet, the ability to derive mini-LMSs directly from in vivo human hearts significantly elevates their potential as they pave the way for a whole new area of expertise in, for example, congenital heart disease and cardiac transplantation medicine. Unpublished data from our lab using pediatric tissue from a tetralogy of Fallot patients already shows great potential, allowing, for the first time, to examine specific biomechanical features and pharmacological testing in tetralogy patients. In addition to that, mini-LMS allow for a fascinating new era of semi-acute dynamic analyses in a patient-/disease-specific setting that was not possible before, and our incorporated setup with real-time optical and electrode mapping on LMSs might provide the ability to better understand the excitation–contraction dynamics in both normal and diseased tissues [22,23], allowing for patient-tailored medicine.

## 5. Conclusions

We developed a novel method to create functional mini-LMSs and outlined their biomechanical characteristics for semi-acute experimentation. Here, we show tissue viability for up to one week before structural changes appear. This method paves the way for innovative translational approaches within, for example, congenital heart diseases and transplantation medicine.

## Figures and Tables

**Table 1:** Baseline biomechanical profile parameters and statistical significance across mini-LMS, medium LMS, and large LMS groups with Kruskal–Wallis H test.

	Mini-LMSs (*n* = 34)	Medium LMSs (*n* = 25)	Large LMSs (*n* = 30)	H	*p*-Value
CD (ms)	501.5 (458.83–560.48)	540 (481.47–611.02)	559.16 (477.98–588.33)	3.379	0.185
Fmax (μN)	1234.14 (583.63–2472.43)	1119.4 (480.81–5004.09)	1666.54 (1273.61–2141.51)	1.953	0.377
TTP (ms)	180.85 (160.67–212.8)	198.28 (175.07–216.28)	217.77 (197.58–232.35)	11.38	0.003
TTR (ms)	334.31 (269.75–402.33)	333.81 (301.71–397.5)	335.39 (287.08–371.57)	1.061	0.589
AUC (μN.s)	372.82 (171.94–879.33)	335.11 (156.39–1576.86)	534.93 (386.14–779.6)	3.843	0.146
+dF/dt (μN/s)	10,397.5 (4501.23–18553.02)	6347.33 (3320.83–26712.99)	10,056.81 (6366.42–15,181.58)	0.543	0.762
-dF/dt (μN/s)	−6088.98 (−11,129.17–3036.83)	−4510 (−20,011.67–2532.17)	−7811.38 (−11,055.67–5497.26)	1.793	0.408

## Data Availability

The raw data supporting the conclusions of this article will be made available by the authors on request.

## Abbreviations

The following abbreviations are used in this manuscript:

LMSs	Living Myocardial Slices
mini-LMSs	Miniaturized LMSs
LV	Left Ventricle
TMDs	Triangular Mounting Devices
BMCCs	Biomimetic Cultivation Chambers
Fmax	Maximum Contraction Force
CD	Contraction Duration
AUC	Area under the Contraction Curve
TTP	Time-to-Peak
TTR	Time-to-Relaxation
AIC	Akaike Information Criterion
IQR	Median and Inter Quartile Range
PBS	Phosphate-Buffered Saline
PFA	Paraformaldehyde
HE	Hematoxylin and Eosin
ICD	Intercalated Discs
FFPE	Formalin-Fixed, Paraffin-Embedded
